# Intradiscal Nucleus Pulposus Allograft Versus Sham Procedure in Patients with Lumbar Discogenic Pain: A Randomized Clinical Trial

**DOI:** 10.3390/jcm15218389

**Published:** 2026-10-28

**Authors:** Paul Verrills, Vahid Mohabbati, James Yu, Matthew Green, Daniel Bates, Harrison Mihailidis, Marc A. Russo

**Affiliations:** 1 Metro Pain Group, Monash House, 271 Clayton Road, Clayton, VIC 3168, Australia; dan.bates@backneckandjoint.com.au (D.B.); harrison.mihailidis@gmail.com (H.M.); 2 Sydney Pain Day Surgery, Suite 3/44-46 Oxford St., Epping, NSW 2121, Australia; director@sydneypaincentre.com; 3 Advanced Spine & Pain, Prince of Wales Private Hospital, Level 7, Suite 24/346 Barker St., Randwick, NSW 2031, Australia; drjamesyu1@gmail.com; 4 PainMedSA, 5 Greenhill Rd, Wayville, SA 5034, Australia; matthew.green@painmedsa.com; 5 Hunter Pain Specialists, 91 Chatham St., Broadmeadow, NSW 2292, Australia; pi@genesisresearchservices.com

**Keywords:** nucleus pulposus, allograft, discogenic, back pain, degenerative disk disease

## Abstract

**Background/Objectives:** Chronic lumbar discogenic pain is a leading cause of disability. We evaluated the efficacy and safety of a single intradiscal injection of nucleus pulposus allograft compared with a sham procedure. **Methods:** Randomized, double-blind, sham-controlled superiority trial at five Australian pain centers. Adults with chronic lumbar discogenic pain were assigned 1:1 to allograft or sham. The prespecified primary endpoint was the proportion of participants with a ≥30% reduction in back pain visual analog scale (VAS) at 6 months. Sham non-responders could cross over to allograft treatment at 3 months; their 3-month values were carried forward to 6 months (last observation carried forward, LOCF). **Results:** A total of 121 participants were randomized (allograft 60, sham 61). At 3 months, the last visit at which both groups were fully observed, 53% (32/60) of allograft and 5% (3/60) of sham participants achieved the back pain responder threshold (difference 48 percentage points, 95% CI 33 to 61; *p* < 0.001). At 6 months, with LOCF for the 55 of 61 sham participants who crossed over, responder rates were 60% (36/60) versus 1.6% (1/61) (difference 58 percentage points, 95% CI 44 to 70; *p* < 0.001); superiority persisted in sensitivity analyses unless ≥45% of crossover participants would have responded untreated. Oswestry Disability Index responder rates were 53% versus 32% at 3 months and 60% versus 31% at 6 months (LOCF). Within 3 months, procedure- or product-related adverse events occurred in 6/60 (10%) allograft and 3/61 (5%) sham participants; two of these events were serious. **Conclusions:** Nucleus pulposus allograft produced large, clinically meaningful reductions in discogenic back pain compared with sham in the fully observed 3-month comparison. Most sham participants crossed over at three months as permitted by the protocol, and their three-month values were carried forward to the 6-month primary endpoint analysis. Registration: NCT06615505.

## 1. Introduction

It has been projected that over 800 million people globally will suffer from low back pain by 2050 [1]. This chronic condition remains the leading cause of disability burden worldwide and its increasing prevalence will continue to exact an economic toll on already fragile healthcare systems. While most cases of new-onset low back pain are self-limiting, recurrence is common, and in a subset of people, low back pain becomes persistently bothersome and disabling [2]. In at least 40% of cases of chronic low back pain, degeneration of the intervertebral disk is the structural culprit and serves as the primary pain generator [3,4].

Degeneration of the intervertebral disk, which commences in the third decade of life, is the antecedent of all subsequent degenerative morphological changes in the lumbar spine and the primary driver of motion segment dysfunction [5,6,7,8,9]. With disk degeneration, the synergy between the inner gelatinous nucleus pulposus and fibrous outer annulus breaks down and the once abundant proteoglycan content that serves to provide substantial load bearing and inhibition of vascular and nerve ingrowth diminishes [10,11,12,13]. Consequently, the loss of intradiscal mechanical integrity due to the decreased ability of the nucleus to bind water under compression precipitates a vicious cycle of advancing degeneration across the entire lumbar motion segment [14,15,16].

The renewed appreciation of the integral role of the intervertebral disk in spinal degeneration has spawned a concerted clinical research effort aimed at developing therapeutic procedures that specifically target the degenerated and painful disk with the goal of preserving disk structure and restoring function [17,18,19,20]. These targeted intradiscal interventions are meant to directly address the underlying pathology utilizing restorative and regenerative products and agents [21,22,23].

Nucleus pulposus allograft is lyophilized, micronized tissue that is reconstituted in sterile saline at the time of the procedure and delivered intradiscally under fluoroscopic guidance. This allogeneic tissue is intended to supplement and replenish the extracellular matrix of the degenerated disk. Two pilot studies of this product showed clinically significant improvements in back pain, back function and quality of life following a single intradiscal procedure over two years of follow-up [24,25,26,27].

Based on the encouraging findings from these feasibility investigations, the current randomized sham-controlled trial was designed and conducted to confirm evidence of the efficacy and safety of intradiscally delivered nucleus pulposus allograft in patients with lumbar discogenic pain. Herein, we present the results of the primary efficacy and safety endpoint analysis of this trial.

## 2. Materials and Methods

### 2.1. Study Design

This was a sham-controlled, double-blind randomized clinical trial conducted at five pain management clinics in Australia. The overall study duration was 24 months. The primary efficacy endpoint was specified a priori at 6 months postprocedure. The selection of 6 months as the primary endpoint aligns with established guidance for chronic pain clinical trials based on the IMMPACT (Initiative on Methods, Measurement, and Pain Assessment in Clinical Trials) recommendations, developed in collaboration with the US Food & Drug Administration [28,29]. All patient-reported outcome measures were collected preprocedure as well as at 1, 3, and 6 months. Additional follow-up will occur at 12 and 24 months postprocedure.

The protocol, approved by the ethics committee and registered before enrollment began, permitted sham participants with less than a 30% reduction in back pain at 3 months to cross over to open-label allograft treatment. This provision was adopted in concert with eligibility requiring failure of at least 3 months of conservative care. An additional 3 months without benefit after a sham procedure was judged to be the longest untreated interval acceptable to participants and investigators. Separately, blinding was expected to be difficult to maintain beyond 3 months in untreated sham participants with chronic pain. A consequence of this design, addressed in the statistical analysis and in the Discussion, is that the 6-month primary endpoint for sham participants who crossed over is based on imputed rather than observed values, whereas the 3-month visit provides a fully observed blinded comparison.

The study was approved by the Bellberry Human Research Ethics Committee (HREC) and all participants provided informed consent. The trial was carried out in accordance with Good Clinical Practice and the principles of the Declaration of Helsinki. The trial was externally monitored by an independent contract research organization and data were managed and analyzed by an independent statistical consulting firm. This trial is reported in accordance with the Consolidated Standards of Reporting Trials (CONSORT) guidelines, and was registered at ClinicalTrials.gov, NCT06615505.

### 2.2. Participants

Patients were eligible for enrollment if they were 22 to 85 years of age with chronic axial low back pain with or without referred non-radicular leg pain of at least 6 months’ duration, and whose pain was unresponsive to 3 months of conservative care. Magnetic resonance imaging (MRI) evidence of disk degeneration (modified Pfirrmann grades of 3–7) was required at 1 or 2 lumbar level(s). Physical examination criteria consisted of a current back pain score of ≥40 to ≤90 mm on a 100 mm visual analog scale (VAS), a back functional impairment score of ≥40 to ≤80 on the Oswestry Disability Index (ODI), intolerance to sitting for >30 min, and positive pain provocation with sustained hip flexion.

Patients were ineligible for this trial if they had a contraindication to MRI or to the sedation/anesthesia protocol. Exclusion criteria also included degenerative changes at >2 lumbar disk levels, previous treatment at the target disk(s), pathological lumbar spine conditions, dynamic instability, grade ≥ 2 spondylolisthesis, spondylitis, Type 3 Modic changes adjacent to the target disk(s), full-thickness annular tears at the target disk(s), and facet arthropathy. Full-thickness annular tears were identified on the screening MRI as a high-intensity zone or linear T2 hyperintensity extending to the outer annulus, focal disruption of the outer annular fibers, or disk material beyond the annular margin; provocative discography was not required and was performed at the discretion of the investigator.

### 2.3. Randomization and Blinding

Participants were randomized to the nucleus pulposus allograft group or the sham control group in a 1:1 ratio via permuted block randomization, stratified by clinical site. Treatment assignments were coded to ensure blinding and were provided via a secure, web-based randomization system.

To protect against selection bias, the randomized treatment allocation was blinded, and treatment assignment was concealed until the day of the procedure. The following individuals were blinded: participants, outcome assessors, clinicians completing physical and neurological examinations, and study coordinators administering patient reported outcome measures and collecting other participant data. The investigator performing the procedure was unblinded.

Participants in the sham control group with less than a 30% reduction in back pain severity were eligible for crossover to intradiscal allograft treatment at the 3-month follow-up visit and unblinded. Participants in the allograft group or sham participants with a 30% or greater reduction in back pain severity were informed that they did not meet the criteria for crossover.

### 2.4. Procedures

Participants in the investigational arm received nucleus pulposus allograft intradiscally using a minimally invasive technique. The nucleus pulposus allograft product, VIA Disc NP (Vivex Biologics, Inc., Miami, FL, USA), was recovered from donated cadaveric disk tissue, lyophilized, and ground to particles that are ≤106 µm in size. The nucleus pulposus particulate was then aliquoted into a volume size of 100 mg (+/− 10%) in a custom mixing system, aseptically sealed and sterilized. Nucleus pulposus allograft was mixed with 2 mL sterile saline (0.9% sodium chloride) and administered into the nucleus pulposus using a 20-gauge cannula under fluoroscopic guidance, utilizing a posterolateral approach to the disk through Kambin’s triangle. A local anesthetic at the site of injection and conscious sedation were used in all cases.

Participants randomized to the control arm received a sham procedure. The procedure was identical to the investigational procedure with the following exception: a 20-gauge cannula was inserted through the skin and muscle of the back but did not penetrate the annulus fibrosus of the intervertebral disk(s). Sham procedures mimicked setup, the procedure itself and the sedation protocol but did not involve an intradiscal injection of either saline or allograft.

Immediately postprocedure all participants were asked to complete a participant treatment survey to deduce the treatment they received: nucleus pulposus allograft, sham or unsure.

### 2.5. Outcomes

Back pain severity was assessed using a standard 100 mm VAS. Back functional impairment was assessed using the ODI. Patient reported outcomes were recorded preprocedure as well as at 1, 3, and 6 months, postprocedure; 12- and 24-month data will be reported separately.

The primary efficacy endpoint of this trial was the between-group comparison of the proportion of participants who achieved a MCID, defined as ≥30% reduction in back pain VAS score from baseline to 6 months [30,31]. We also computed responder rates for substantial clinical benefit (SCB) defined as ≥50% reduction in back pain VAS score and the patient acceptable symptom state (PASS) defined as a final back pain VAS score ≤ 30 mm. Similarly, we computed the MCID (≥30% reduction), SCB (≥50% reduction) and PASS (≤20 final score) responder rates for the ODI.

All adverse events were captured in real time and evaluated by the site investigator with respect to severity and relatedness. Safety analysis of cumulative adverse events by study group was conducted at the 3-month follow-up interval, prior to sham crossover. Both procedure- and product-related adverse events were captured and judged as not, possibly or definitely related to the occurrence of the event. All adverse events were independently adjudicated by the trial-designated medical monitor.

### 2.6. Statistical Analyses

This was a planned superiority trial. The sample size was determined to ensure an 80% probability of identifying a meaningful between-group difference in the proportion of participants achieving a MCID in the back pain VAS score at 6 months. The expected success rate in the sham control group was estimated at 32.5% and 60% in the allograft group. To maintain a 5% risk of Type I error, it was calculated that 55 participants would be required in each study group (110 total participants).

Estimand. The primary estimand was the difference between treatment groups in the proportion of randomized participants achieving a ≥30% reduction in back pain VAS from baseline at 6 months, had sham participants not crossed over to allograft treatment. All randomized participants were analyzed in their allocated groups using an intention-to-treat approach. Missing data. For sham participants who crossed over at 3 months (participants), the 3-month value was carried forward to 6 months (last observation carried forward, LOCF), as prespecified in the statistical analysis plan; this assumes no change in untreated sham non-responders between 3 and 6 months. Four sham participants missed the 1-month visit, one sham participant was lost to follow-up before the 3-month visit, and one allograft participant withdrew after the 3-month visit; in each case the most recent prior assessment was carried forward, which classifies the sham participants as non-responders. The 3-month comparison, at which 60 of 60 allograft and 60 of 61 sham participants were observed, is reported as the fully observed randomized comparison. Sensitivity analyses for the 6-month endpoint were (i) a tipping-point analysis in which the number of sham responders was increased until superiority was lost and (ii) a scenario analysis assigning the crossover participants 6-month responder rates from 0% to 60%. Inference. Responder proportions were compared with Fisher’s exact test and the difference in proportions is reported with Newcombe hybrid-score 95% confidence intervals; Cohen’s h is given as a standardized effect size. Continuous change scores were compared with the two-sided Wilcoxon rank-sum test, prespecified because of expected skewness; between-group differences are reported as differences in means with 95% CIs, and Cohen’s d was calculated as the difference in mean change divided by the pooled standard deviation of the change scores. Effect size thresholds were 0.2 (small), 0.5 (medium), 0.8 (large), and 1.3 (very large) [32].

Prespecified exploratory subgroup analyses of the 6-month back pain responder endpoint within the allograft group compared age (<52 vs. ≥52 years; median), BMI (<28 vs. ≥28 kg/m^2^; median), sex, number of levels treated (1 vs. 2), maximum modified Pfirrmann grade (3–5 vs. 6–7) and maximum Modic change (0 vs. 1–2) using Fisher’s exact test, two-tailed. No adjustment was made for multiplicity in secondary or exploratory analyses.

A cumulative proportion of responders’ analysis (CPRA) was used to display the proportion of participants achieving various levels of treatment response for back pain VAS across all possible cutoff points [33]. Evaluation of treatment assignment blinding fidelity utilized the method of Petroff et al. [34].

## 3. Results

Between 4 October 2024 and 30 July 2025, 210 adults were assessed for eligibility, of whom 89 failed to meet one or more entry criteria. 121 adults were enrolled and randomly assigned to receive either a single 100 mg dose of nucleus pulposus allograft (60 participants, 92 disks treated) or a sham procedure (61 participants, 91 disks treated; Figure 1). Baseline characteristics by study group are provided in Table 1.

Visit attendance was 118 of 121 (97.5%) at 1 month, 120 (99%) at 3 months and 119 (98%) at 6 months (Figure 1, Table S1). In the allograft group all 60 participants were assessed at 1 and 3 months and 59 at 6 months. In the sham group 57 participants were assessed at 1 month and 60 at 3 months. At 3 months, 57 sham participants had less than a 30% reduction in back pain and were eligible to cross over; 55 did so and were unblinded and treated after the 3-month visit, and 2 declined. Five of the remaining six sham participants (three responders and two who declined crossover; one was lost to follow-up) continued untreated and were assessed at 6 months. Consequently 56 of 61 (92%) sham values at the 6-month primary endpoint are carried forward (55 three-month values for crossover participants and one earlier value for the participant lost to follow-up, counted as a non-responder), and 1 of 60 allograft values is a 3-month value carried forward.

At 3 months, the last visit at which both groups were fully observed under blinded conditions, the back pain MCID (≥30%) was achieved by 32 of 60 allograft participants (53.3%) and 3 of 60 sham participants (5.0%) (difference 48.3 percentage points, 95% CI 33.2 to 60.8; *p* < 0.001; Cohen’s h 1.19; number needed to treat 2.1) (Table 2). Counting the one sham participant lost to follow-up as a non-responder gave the same result (3 of 61, 4.9%). Mean back pain VAS had decreased by 24.8 mm (SD 26.6) in the allograft group and 2.9 mm (SD 12.9) in the sham group (difference −21.9 mm, 95% CI −29.4 to −14.4; *p* < 0.001; d = 1.05). For ODI, 3-month MCID responder rates were 53.3% (32/60) and 31.7% (19/60) (difference 21.7 percentage points, 95% CI 4.0 to 37.5; *p* = 0.03), and the mean change differed by −10.5 points (95% CI −17.1 to −4.0; *p* = 0.002; d = 0.57).

The prespecified 6-month primary endpoint, analyzed with LOCF for the 55 sham participants who crossed over, was met: back pain MCID responder rates were 60.0% (36 of 60) in the allograft group and 1.6% (1 of 61) in the sham group (difference 58.4 percentage points, 95% CI 43.9 to 69.9; *p* < 0.001; h = 1.52) (Table 2). SCB in back pain was achieved in 53% of allograft participants (32 of 60) and 1.6% of sham participants (1 of 61) (difference 51.7 percentage points, 95% CI 38.7 to 64.7, *p* < 0.001; h = 1.4). Twenty-nine (48.3%) allograft participants and 1 (1.6%) sham participant achieved the 6-month PASS (difference 46.7 percentage points, 95% CI 33.7 to 59.7, *p* < 0.001; h = 1.3). Because the 3-month values of crossover participants were carried forward, the sham estimate at 6 months reflects observed data for 5 participants and imputed data for 56 and should be read as the result expected if untreated sham non-responders remained constant between 3 and 6 months. Of the three sham participants who were responders at 3 months and remained untreated, one remained a responder at 6 months. In sensitivity analyses (Table S2, Figure S1), the between-group difference remained statistically significant with up to 25 sham responders in total (24 of the 55 crossover participants, 44%; *p* = 0.046) and was lost at 26 (25 crossover participants, ≥45%; *p* = 0.07); under the sham responder rates observed at 1 month (28%) or assumed at trial planning (32.5%), the differences would be 34 (95% CI 16 to 49) and 29 (95% CI 11 to 44) percentage points respectively.

Figure 2 provides a responder rate scatterplot by study group of each participant’s individual back pain VAS values at 1, 3 and 6 months postprocedure. Figure 3 shows the CPRA diagram at the 1- and 3-month follow-up intervals by study group, which visually contrasts substantial improvements in the proportion of patients achieving reductions in back pain severity in the allograft group and the sham crossover group, but not in the original sham group. Figure 4 provides the therapeutic trajectories for the allograft, sham and sham crossover groups through 6 months of follow-up.

For ODI, the 6-month LOCF MCID responder rates were 60% (36 of 60 participants) and 31.1% (19 of 61 participants) for allograft and sham participants, respectively (difference 28.9 percentage points, 95% CI 11.9 to 45.8, *p* = 0.001; h = 0.6). The between-group mean difference in change from baseline was 14.9 (95% CI 9.2 to 20.7, *p* < 0.001; d = 0.9). ODI SCB was achieved in 46.7% (28 of 60) and 13.1% (8 of 61) of allograft and sham participants, respectively (difference 33.6 percentage points, 95% CI 17.5 to 47.5; *p* < 0.001; h = 0.8), and the ODI PASS in 36.7% (22 of 60) and 9.8% (6 of 61) (difference 26.8 percentage points, 95% CI 11.9 to 40.5; *p* < 0.001; h = 0.7).

In exploratory analyses within the allograft group, 6-month back pain responder rates did not differ significantly by age, BMI, sex, number of treated levels, modified Pfirrmann grade or Modic change (Figure 5). These analyses were not powered to detect subgroup differences and do not establish that the treatment effect is homogeneous.

Evaluation of the results of the participant treatment survey found no significant differences between study groups in the ability to deduce treatment group assignment, with 61% of allograft and 72% of sham participants reporting that they were unsure of their treatment assignment (*p *= 0.29).

Within the prespecified 3-month controlled safety window, adverse events judged related to the procedure or the product occurred in 6 of 60 allograft participants (10.0%; 6 events) and 3 of 61 sham participants (4.9%; 3 events) (Table S3). Procedure-related events: allograft 5 (2 definitely, 3 possibly related: back pain × 2, arthralgia, pain, sciatica); sham 3 (all possibly related: back pain × 2, maculopapular rash). Product-related events: allograft 4 (1 definitely, 3 possibly related: back pain, arthralgia, pain, flank pain). All related events in both groups occurred within 30 days of the procedure. Two allograft participants had serious related events: one was hospitalized for a back pain flare after the procedure, judged both procedure- and product-related, and resolved with conservative management without sequelae; the second reported severe back pain that was procedure-related and also resolved with conservative management without sequelae. No sham participant had a serious related event. Serious adverse events of any cause occurred in four allograft (6.7%) and three sham (4.9%) participants.

## 4. Discussion

In this randomized, double-blind, sham-controlled trial, a single intradiscal injection of nucleus pulposus allograft produced substantially greater relief of discogenic low back pain than a sham procedure. In the fully observed, blinded comparison at 3 months, 53% of allograft and 5% of sham participants achieved a clinically important reduction in back pain, a difference of 48 percentage points corresponding to a number needed to treat of about 2. The prespecified 6-month primary endpoint was also met; however, because 55 of 61 sham participants crossed over to allograft treatment at 3 months as permitted by protocol, the 6-month sham estimate rests on carrying their 3-month values forward, and the size of the 6-month effect therefore depends on the assumption that untreated sham non-responders would not have improved between 3 and 6 months. Almost one-half of allograft participants reached the patient acceptable symptom state at 6 months, a threshold that distinguishes patients who feel well from those who merely feel better [35].

The rationale for nucleus pulposus allograft comes from the accepted understanding that the healthy nucleus functions as a hydrated, proteoglycan-rich tissue [36,37], and that its depletion with degeneration contributes to discogenic pain. Supplementing the degenerated disk with homologous nucleus tissue is intended to replenish this matrix.

The sham response in this trial was initially typical of procedural trials in back pain [38,39]—28% of sham participants achieved the back pain MCID at 1 month—but it was not sustained: at 3 months 5% were responders, 38% reported no improvement over baseline, and the median percentage change was 0%. Of the three sham responders at 3 months who remained untreated, one remained a responder at 6 months. By contrast, 32% of sham participants achieved the ODI responder threshold at 3 months, so the attenuated placebo response concerned pain intensity rather than self-reported function. We found no evidence that sham participants identified their allocation more readily than allograft participants, and most participants in both groups were unsure of their assignment.

Why the pain response to sham dissipated cannot be determined from this trial. Two features of the design offer candidate explanations: the eligibility criteria required sitting intolerance and a positive sustained hip-flexion test in addition to imaging criteria, which may have enriched the sample for mechanically determined discogenic pain and excluded patients whose symptoms fluctuate; and the sham procedure delivered no stimulus to the disk itself, whereas trials using intradiscal saline or anesthetic as control deliver an intervention that may itself alter symptoms. Two earlier sham-controlled trials of intradiscal procedures that also avoided an intradiscal control stimulus likewise reported little placebo response [40,41].

We found no evidence that sham participants in the current trial were able to deduce their treatment assignment postprocedure more easily than participants assigned to the allograft group. In fact, most participants, irrespective of study group, were unsure of their treatment assignment, suggesting a lack of confidence in choosing one possibility over the other. Maintenance of blinding to treatment assignment was likely assured due to the uniform use of conscious sedation during all procedures.

We noted that the group of sham participants that crossed over to receive the allograft procedure open-label experienced a degree of symptom improvement similar to that of the group of participants in the original blinded allograft arm of the trial (Figure 4). For example, the back pain MCID was achieved in 56% (31 of 55 participants) of sham crossover participants compared with 53% (32 of 60 participants) of original allograft participants at the same 3-month follow-up interval. The response of the unblinded crossover participants was therefore consistent with that of the blinded allograft arm.

Within the 3-month controlled window, procedure- or product-related adverse events occurred in 10% of allograft and 5% of sham participants, and two allograft participants had serious related events, both resolving with conservative management. All related events occurred within the first month. With 60 treated participants, the trial can exclude only common serious harms—an event not observed in 60 participants has an upper 95% confidence limit of approximately 5%—and the controlled comparison was limited to 3 months. It should be noted that a recent product-safety analysis found a low frequency of adverse events and product complaints [42].

These results apply to the population enrolled: adults with at least 6 months of axial low back pain unresponsive to 3 months of conservative care, baseline VAS 40–90 mm and ODI 40–80, MRI degeneration (modified Pfirrmann 3–7) at no more than two lumbar levels, sitting intolerance and a positive sustained hip-flexion test, without instability, spondylolisthesis of grade 2 or more, Modic type 3 change, a full-thickness annular tear or facet arthropathy. Forty-two percent of screened patients did not meet these criteria and were ineligible. Thus, the findings should not be extrapolated to nonspecific chronic low back pain or to degenerative disk disease in general.

Some limitations of this study should be addressed. First, the protocol permitted sham non-responders to cross over at 3 months, and 55 of 61 did so; as a result the prespecified 6-month primary endpoint compares observed allograft outcomes with sham outcomes that are imputed for 92% of participants (56 of 61). Intention to treat using last observation carried forward assumes that untreated sham non-responders would not have improved between 3 and 6 months; this is consistent with the declining sham response observed through 3 months and with the observation that four of the five untreated sham participants assessed at 6 months were non-responder, although no statistical method can recover a fully observed six-month randomized comparison. The 3-month comparison is the least assumption-dependent estimate of the randomized treatment effect, and the sensitivity analyses indicate how far the 6-month conclusion depends on the unobserved outcomes. Second, the controlled safety comparison was limited to 3 months and 60 treated participants. Third, the trial did not include imaging or biomechanical outcomes. Fourth, full-thickness annular tears were excluded on MRI, which has limited sensitivity for annular fissures. Fifth, the eligibility criteria restrict generalizability, as discussed above. Longer-term efficacy, including the need for subsequent spinal procedures, will be reported from the 12- and 24-month follow-up.

## 5. Conclusions

In patients with chronic lumbar discogenic pain selected by clinical and imaging criteria, a single intradiscal injection of nucleus pulposus allograft produced large, clinically meaningful improvements in back pain and function compared with a sham procedure in the fully observed 3-month randomized comparison. The prespecified 6-month primary endpoint was met. With most sham participants crossing over at 3 months, the six-month estimate depends on imputation, and the conclusion of 6-month superiority was robust across a wide range of assumptions about the unobserved sham outcomes. Related adverse events were infrequent within the initial 3-month follow-up interval, although the trial was not large enough to characterize uncommon serious harms. Longer-term follow-up of this cohort will assess durability in the blinded allograft arm and the reproducibility of the treatment effect in the unblinded crossover participants.

## Figures and Tables

**Figure 1:**
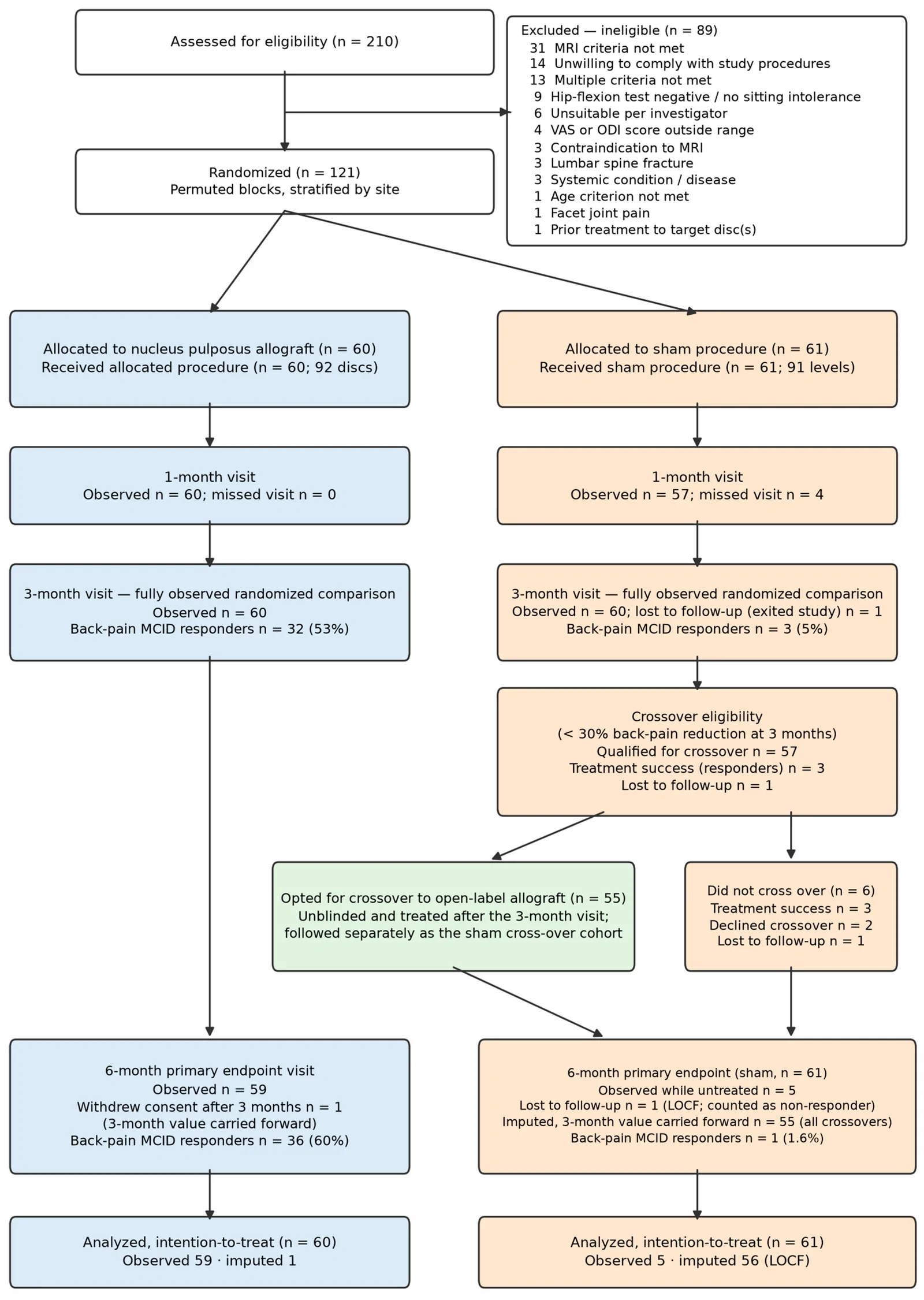
Participant flow adapted for the protocol-specified 3-month sham crossover, showing enrollment, allocation, follow-up at each visit, crossover eligibility and uptake, and the observed and imputed composition of each analysis set. LOCF, last observation carried forward.

**Figure 2:**
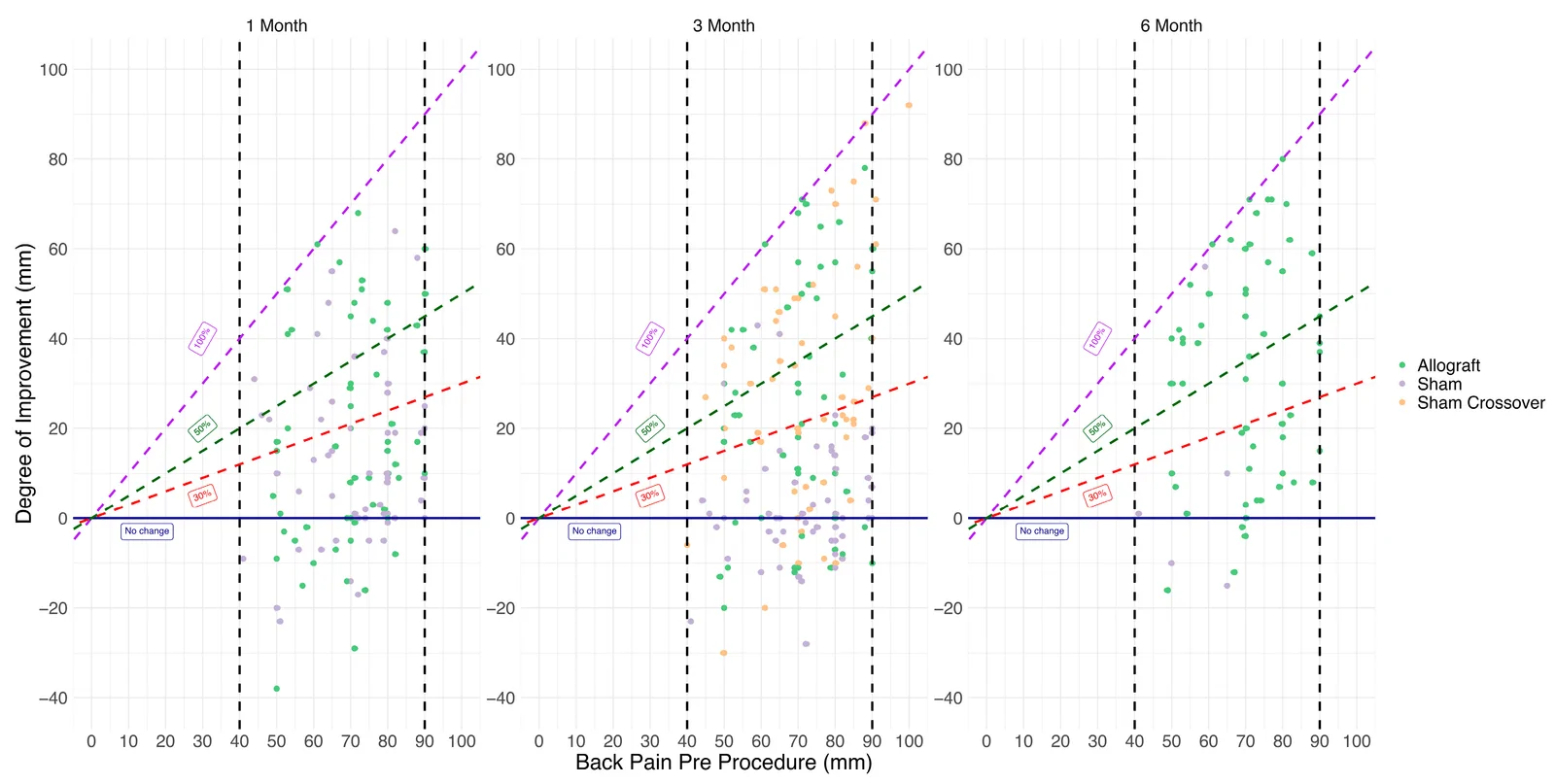
Scatterplot illustrating the relationship between pre-treatment back pain severity and absolute degree of pain improvement at 1, 3 and 6 months. Data are presented in mm, with the diagonal lines representing the percentage change success thresholds for the minimal clinically important difference (MCID, 30%), substantial clinical benefit (SCB, 50%) and complete pain relief (100%). Vertical lines demarcate the initial back pain VAS eligibility criteria.

**Figure 3:**
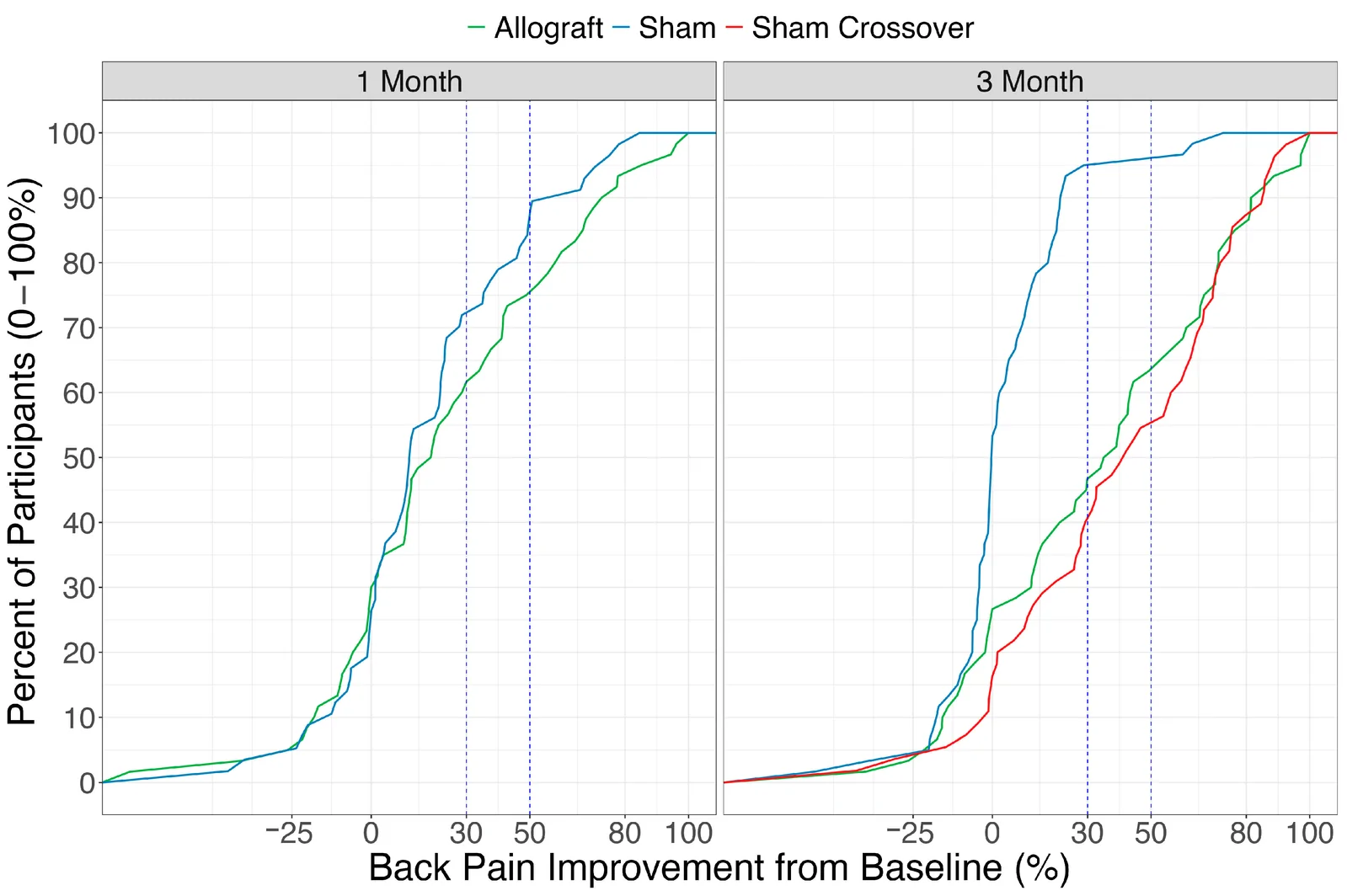
Cumulative proportion of responders analysis (CPRA) plots at 1 and 3 months with dotted vertical lines representing the MCID (30%) and SCB (50%) success thresholds. The corresponding 3-month median percentage improvements from baseline were 37% (IQR, 0% to 68.4%), 33% (IQR, 10.4% to 70.0%), and 0% (IQR, −4.8% to 11.6%) for allograft, sham crossover and sham participants, respectively. Allograft (green), sham (blue) and sham crossover (red).

**Figure 4:**
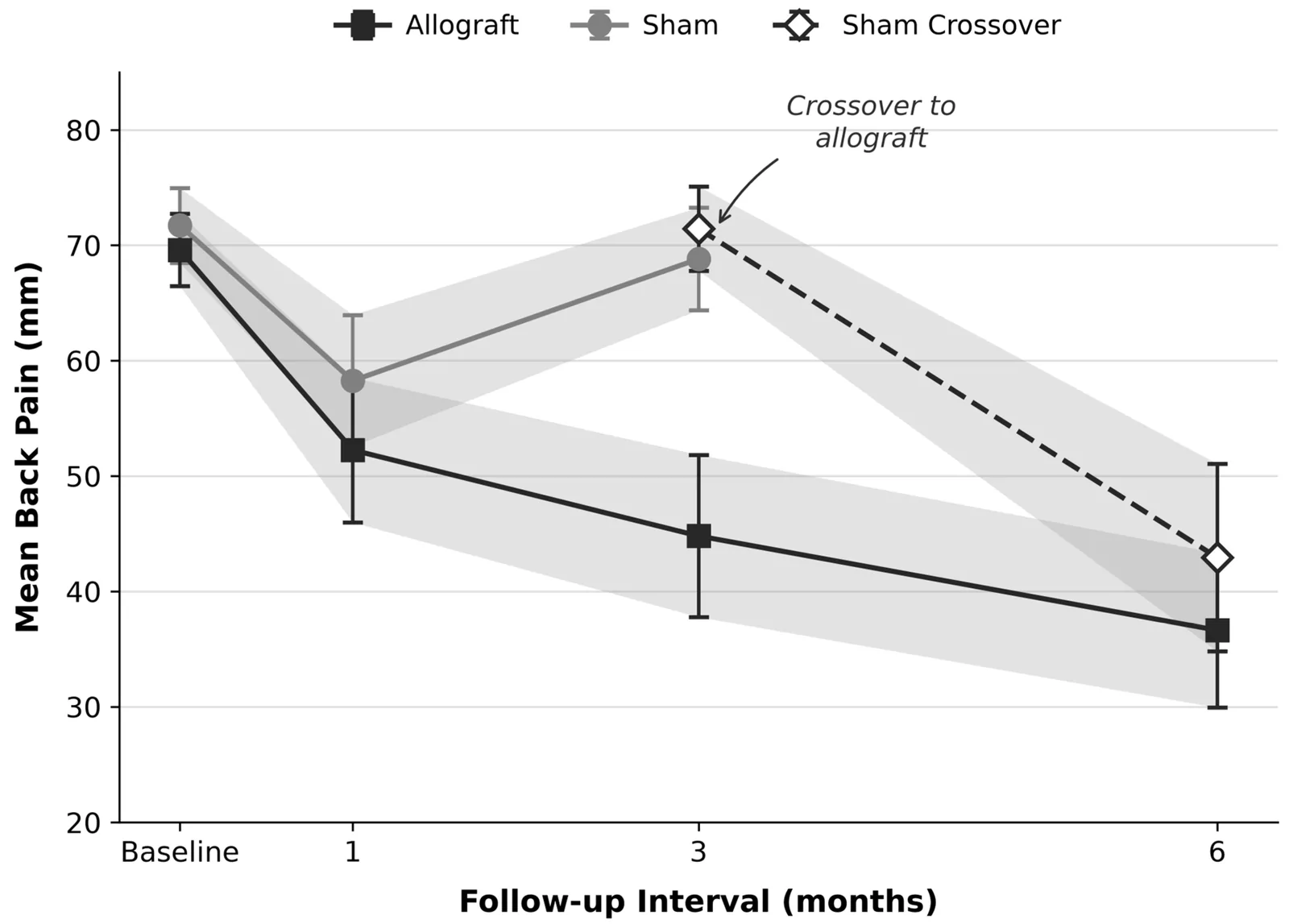
Line graph illustrating mean values (95% CIs) at each follow-up interval for allograft, sham, and sham crossover participants.

**Figure 5:**
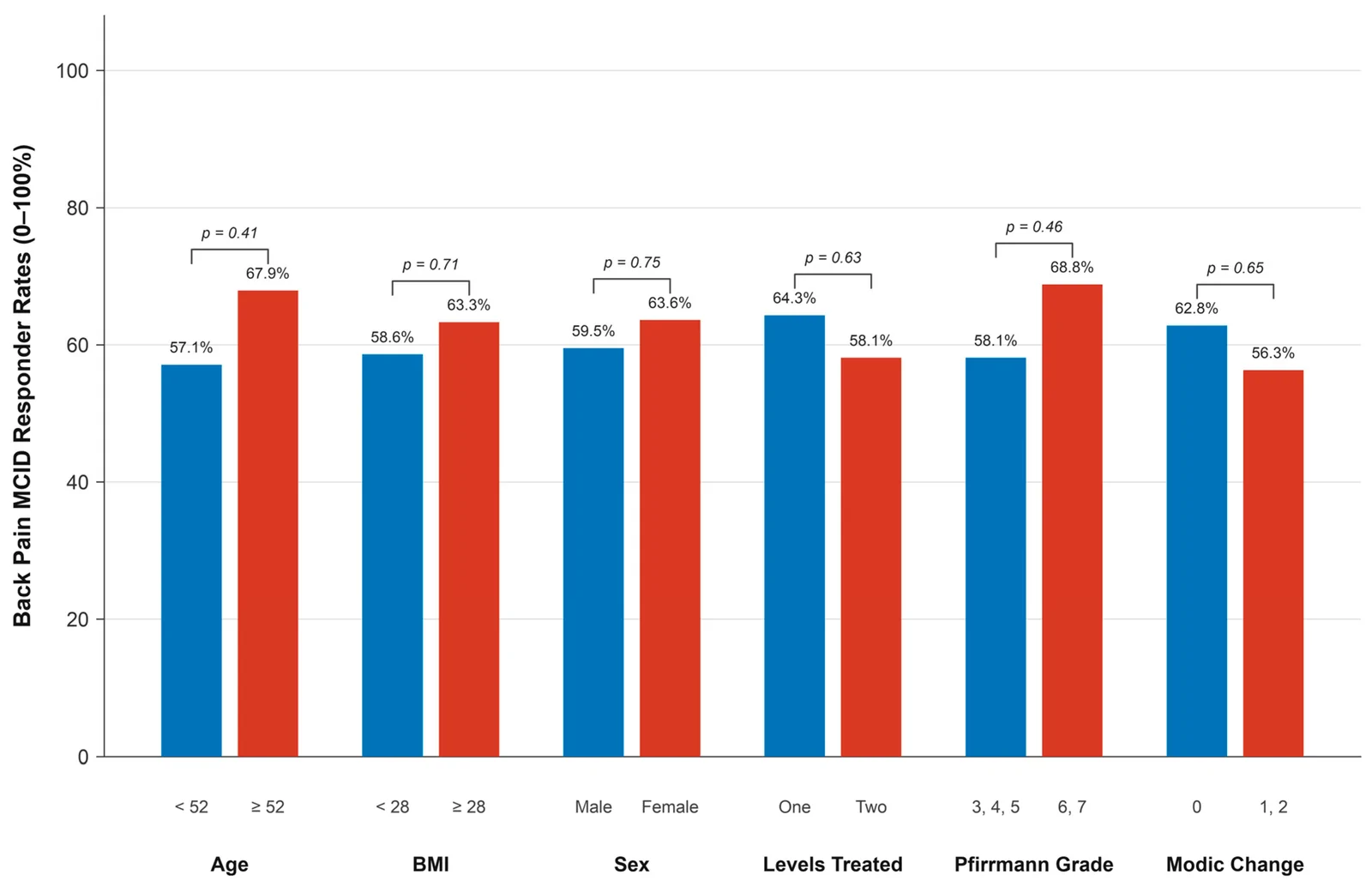
Bar graphs showing subgroup comparisons of 6-month back pain MCID responder rates for the nucleus pulposus allograft participants. Demarcations for age and body mass index (BMI) based on the median group value.

**Table 1:** Baseline characteristics of the intention-to-treat population.

	Nucleus Pulposus Allograft (n = 60)	Sham Control (n = 61)
**Sex**		
Male	37 (62)	38 (62)
Female	23 (38)	23 (38)
Age (years)	52 (13)	51 (14)
BMI (kg/m^2^)	28 (5)	28 (5)
**Number Levels Treated**		
1	28 (47)	31 (51)
2	32 (53)	30 (49)
**Pfirrmann Grade (1st level)**		
3	14 (23)	14 (23)
4	25 (42)	20 (33)
5	7 (12)	10 (16)
6	6 (10)	8 (13)
7	8 (13)	9 (15)
**Pfirrmann Grade (2nd level)**		
3	8 (13)	4 (7)
4	9 (15)	11 (18)
5	7 (12)	6 (10)
6	7 (12)	5 (8)
7	1 (2)	4 (7)
not treated	28 (47)	31 (51)
**Modic Changes (1st level)**		
0	47 (78)	44 (72)
1	7 (12)	9 (15)
2	6 (10)	8 (13)
**Modic Changes (2nd level)**		
0	24 (40)	25 (41)
1	1 (2)	4 (7)
2	7 (12)	1 (2)
not treated	28 (47)	31 (51)
**Level Treated (1st level)**		
L1/L2	0 (0)	1 (2)
L2/L3	0 (0)	4 (7)
L3/L4	1 (2)	3 (5)
L4/L5	30 (50)	30 (49)
L5/S1	29 (48)	23 (38)
**Level Treated (2nd level)**		
L1/L2	0 (0)	0 (0)
L2/L3	2 (3)	2 (3)
L3/L4	7 (12)	5 (8)
L4/L5	12 (20)	7 (12)
L5/S1	11 (18)	16 (26)
not treated	28 (47)	31 (51)
Back Pain VAS (mm)	70 (12)	72 (13)
ODI (%)	51 (12)	51 (11)

Data are n (%), mean (SD), or n/N (%). BMI, body mass index; VAS, visual analog scale; ODI, Oswestry Disability Index.

**Table 2:** Back pain and Oswestry Disability Index outcomes at 3 and 6 months by study group, with data status.

Outcome	Visit	Allograft	Sham	Difference (95% CI)	*p*	Data Status
Back pain VAS MCID (≥30%)	1 month	24/60 (40.0%)	16/57 (28.1%)—observed	11.9 (−5.1 to 29.0)	0.17	Observed
Back pain VAS MCID (≥30%)	3 months	32/60 (53.3%)	3/60 (5.0%)—observed	48.3 (33.2 to 60.8)	<0.001	Fully observed (randomized comparison)
Back pain VAS MCID (≥30%)	6 months (primary)	36/60 (60.0%)	1/61 (1.6%)—56 imputed by LOCF	58.4 (43.9 to 69.9)	<0.001	LOCF (56 of 61 sham values imputed); prespecified primary analysis
Back pain VAS, change from baseline (mm)	3 months	−24.8 (26.6)	−2.9 (12.9)—observed	−21.9 (−29.4 to −14.4); d = 1.05	<0.001	Fully observed (randomized comparison)
Back pain VAS, change from baseline (mm)	6 months (primary)	−32.7 (24.2)	−2.2 (11.9)—56 imputed by LOCF	−30.5 (−37.3 to −23.7); d = 1.60	<0.001	LOCF (56 of 61 sham values imputed)
ODI MCID (≥30%)	3 months	32/60 (53.3%)	19/60 (31.7%)—observed	21.7 (4.0 to 37.5)	0.03	Fully observed (randomized comparison)
ODI MCID (≥30%)	6 months (primary)	36/60 (60.0%)	19/61 (31.1%)—56 imputed by LOCF	28.9 (11.1 to 44.2)	0.001	LOCF (56 of 61 sham values imputed)
ODI, change from baseline (points)	3 months	−18.4 (17.6)	−7.8 (15.3)—observed	−10.5 (−17.1 to −4.0); d = 0.57	0.002	Fully observed (randomized comparison)
ODI, change from baseline (points)	6 months (primary)	−22.8 (16.6)	−7.5 (15.4)—56 imputed by LOCF	−14.9 (−20.7 to −9.2); d = 0.9	<0.001	LOCF (56 of 61 sham values imputed)

Responder differences are in percentage points with Newcombe hybrid-score 95% confidence intervals; continuous differences are differences in mean change with 95% CIs and Cohen’s d. *p* values are from Fisher’s exact test (responders) and the two-sided Wilcoxon rank-sum test (continuous outcomes). One-month values are observed-case (four sham participants missed the visit). At 3 months the sham participant lost to follow-up is excluded (3/61 if counted as a non-responder). At 6 months sham values are carried forward for the 55 crossover participants and for the participant lost to follow-up (56 of 61, 92%). LOCF, last observation carried forward; MCID, minimal clinically important difference (≥30% improvement); ODI, Oswestry Disability Index; VAS, visual analog scale.

## Data Availability

The datasets used and/or analyzed during the current study are available from the corresponding author upon reasonable request.

## Supplementary Materials

The following supporting information can be downloaded at: https://www.mdpi.com/article/10.3390/jcm15218389/s1, Figure S1: Difference in 6-month back pain responder rate (allograft minus sham) as a function of the assumed responder rate among the 55 sham participants who crossed over at 3 months; Table S1: Participant disposition by study group and visit: observed, lost to follow-up, crossed over, and imputed values; Table S2: Sensitivity of the 6-month primary endpoint to assumptions about the 6-month outcome of the 55 sham participants who crossed over at 3 months; Table S3: Adverse events by category and study group.

## Abbreviations

The following abbreviations are used in this manuscript:

BMI	Body Mass Index
CPRA	Cumulative Percentage of Responders’ Analysis
HREC	Human Research Ethics Committee
IMMPACT	Initiative on Methods, Measurement, and Pain Assessment in Clinical Trials
IQR	Interquartile Range
ITT	Intention To Treat
LOCF	Last Observation Carried Forward
MCID	Minimal Clinically Important Difference
MRI	Magnetic Resonance Imaging
ODI	Oswestry Disability Index
PASS	Patient Acceptable Symptom State
SCB	Substantial Clinical Benefit
VAS	Visual Analog Scale
