# Supplementary material for: Intradiscal Nucleus Pulposus Allograft Versus Sham Procedure in Patients with Lumbar Discogenic Pain: A Randomized Clinical Trial
Source: J Clin Med. 2026 Oct 28;15(21):8389. doi: 10.3390/jcm15218389 (PMC13763728; doi:10.3390/jcm15218389)
Supplement: Supplementary file 1 [file jcm-15-08389-s001.zip › jcm-4581612 Figure S1.pdf]

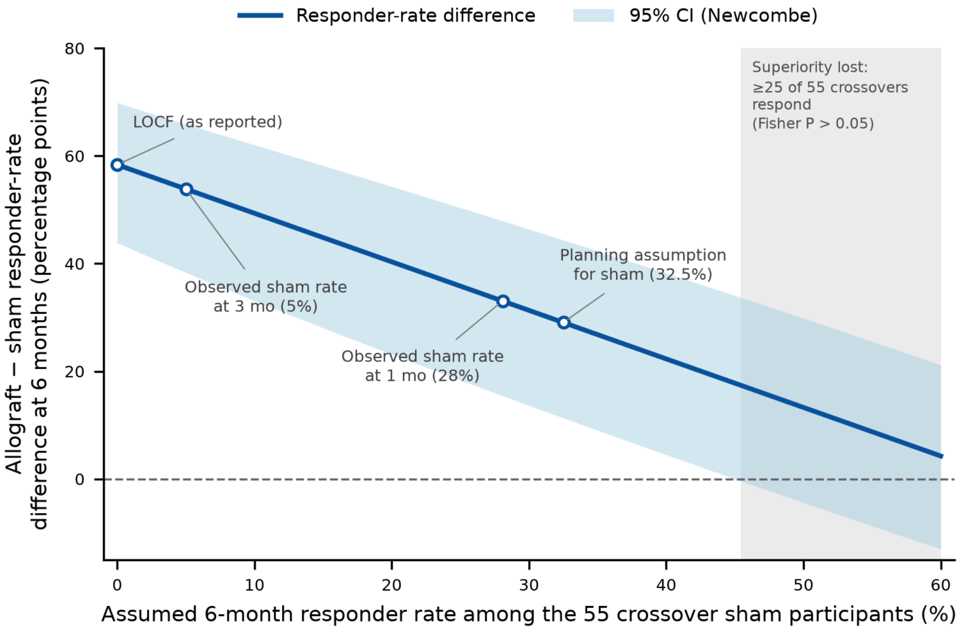

Figure S1. Difference in 6-month back-pain responder rate (allograft minus sham) as a function of the assumed responder rate among the 55 sham participants who crossed over at 3 months. Shaded band, Newcombe 95% CI; grey region, assumptions under which the two-sided Fisher exact P exceeds 0.05. Reference points: LOCF as reported (0%), observed sham rates at 3 months (5%) and 1 month (28%), and the 32.5% sham response assumed in the sample-size calculation.
