# Supplementary material for: Intradiscal Nucleus Pulposus Allograft Versus Sham Procedure in Patients with Lumbar Discogenic Pain: A Randomized Clinical Trial
Source: J Clin Med. 2026 Oct 28;15(21):8389. doi: 10.3390/jcm15218389 (PMC13763728; doi:10.3390/jcm15218389)
Supplement: Supplementary file 1 [file jcm-15-08389-s001.zip › jcm-4581612 Table S1.pdf]

Table S1. Participant disposition by study group and visit: observed, lost to follow-up, crossed over, and imputed values.

Arm	Visit	Observed (untreated per allocation)	Lost to follow-up / missed visit (not crossed over)	Crossed over before visit	Imputed (LOCF)	Imputation source
Allograft (n=60)	1 month	60	0	0	0	—
Allograft (n=60)	3 months	60	0	0	0	—
Allograft (n=60)	6 months (primary)	59	1	0	1	1 withdrew consent after 3 months: 3-month value carried forward
Sham (n=61)	1 month	57	4	0	4	Baseline value carried forward (4)
Sham (n=61)	3 months	60	1	0	1	1 lost to follow-up (exited study): prior value carried forward, counted as non-responder
Sham (n=61)	6 months (primary)	5	1	55	56	3-month value carried forward for all 55 crossover participants; 1 lost to follow-up counted as non-responder

LOCF, last observation carried forward. Sham participants who crossed over at 3 months have their 3-month value carried forward to 6 months; the sham participant lost to follow-up before the 3-month visit is counted as a non-responder at every subsequent visit.
