# Supplementary material for: Intradiscal Nucleus Pulposus Allograft Versus Sham Procedure in Patients with Lumbar Discogenic Pain: A Randomized Clinical Trial
Source: J Clin Med. 2026 Oct 28;15(21):8389. doi: 10.3390/jcm15218389 (PMC13763728; doi:10.3390/jcm15218389)
Supplement: Supplementary file 1 [file jcm-15-08389-s001.zip › jcm-4581612 Table S2.pdf]

Table S2. Sensitivity of the 6-month primary endpoint to assumptions about the 6-month outcome of the 55 sham participants who crossed over at 3 months.

Scenario	Assumed responder rate among crossover participants	Sham responders, n/61 (%)	Difference, percentage points	95% CI	Fisher P	NNT	Cohen h
LOCF / 12-wk status maintained (as reported)	0.0%	1/61 (1.6%)	58.4	43.9 to 69.9	<0.001	1.7	1.52
Observed sham 12-wk responder rate (3/60 = 5%)	5.0%	4/61 (6.6%)	53.4	37.9 to 65.5	<0.001	1.9	1.25
Observed 1-month sham (placebo) responder rate (16/57 = 28%)	28.1%	16/61 (26.2%)	33.8	16.2 to 48.6	<0.001	3.0	0.7
Protocol planning assumption for sham (32.5%)	32.5%	19/61 (31.1%)	28.9	11.1 to 44.2	0.002	3.5	0.59
Tipping point (39%)	39%	24/61 (39.3%)	20.7	2.9 to 36.7	0.029	4.8	0.42
Crossovers respond at allograft 12-wk rate (32/60 = 53%)	53.3%	30/61 (49.2%)	10.8	-6.8 to 27.5	0.275	9.2	0.22

Allograft responders fixed at 36 of 60. The six sham participants who did not cross over contribute their observed status (1 responder). Superiority (two-sided Fisher $P < 0.05$) is retained up to 25 sham responders in total (24 crossover responders, 44%) and lost at 26 (25 crossover responders, 45%). NNT, number needed to treat.
