# Supplementary material for: Intradiscal Nucleus Pulposus Allograft Versus Sham Procedure in Patients with Lumbar Discogenic Pain: A Randomized Clinical Trial
Source: J Clin Med. 2026 Oct 28;15(21):8389. doi: 10.3390/jcm15218389 (PMC13763728; doi:10.3390/jcm15218389)
Supplement: Supplementary file 1 [file jcm-15-08389-s001.zip › jcm-4581612 Table S3.pdf]

Table S3. Adverse events by category and study group.

	Allograft (N = 60)			Sham (N = 61)			Sham cross-over (N = 55)		
**Category**	**Events**	**Participants**	**%**	**Events**	**Participants**	**%**	**Events**	**Participants**	**%**
Adverse Events									
All	46.0	29.0	48.3	31	22	36.1	31.0	20.0	36.4
Product-related>	4.0	4.0	6.7	—	—	—	9.0	7.0	12.7
Product-related - Definitely	1.0	1.0	1.7	—	—	—	1.0	1.0	1.8
Product-related - Possibly	3.0	3.0	5.0	—	—	—	8.0	6.0	10.9
Product-related - Unknown	0.0	0.0	0.0	—	—	—	0.0	0.0	0.0
Product-related - Not Related	42.0	26.0	43.3	—	—	—	22.0	15.0	27.3
Procedure-related>	5.0	5.0	8.3	3	3	4.9	9.0	7.0	12.7
Procedure-related - Definitely	2.0	2.0	3.3	0	0	0	3.0	3.0	5.5
Procedure-related - Possibly	3.0	3.0	5.0	3	3	4.9	6.0	5.0	9.1
Procedure-related - Unknown	0.0	0.0	0.0	0	0	0	0.0	0.0	0.0
Procedure-related - Not Related	41.0	27.0	45.0	28	21	34.4	22.0	15.0	27.3
Product- or procedure-related>	6.0	6.0	10.0	3	3	4.9	9.0	7.0	12.7
Serious Adverse Events									
All	4.0	4.0	6.7	3	3	4.9	5.0	5.0	9.1

Product-related>	1.0	1.0	1.7	—	—	—	1.0	1.0	1.8
Product-related - Definitely	1.0	1.0	1.7	—	—	—	0.0	0.0	0.0
Product-related - Possibly	0.0	0.0	0.0	—	—	—	1.0	1.0	1.8
Product-related - Unknown	0.0	0.0	0.0	—	—	—	0.0	0.0	0.0
Product-related - Not Related	3.0	3.0	5.0	—	—	—	4.0	4.0	7.3
Procedure-related>	2.0	2.0	3.3	0	0	0	1.0	1.0	1.8
Procedure-related - Definitely	1.0	1.0	1.7	0	0	0	1.0	1.0	1.8
Procedure-related - Possibly	1.0	1.0	1.7	0	0	0	0.0	0.0	0.0
Procedure-related - Unknown	0.0	0.0	0.0	0	0	0	0.0	0.0	0.0
Procedure-related - Not Related	2.0	2.0	3.3	3	3	4.9	4.0	4.0	7.3
Product- or procedure-related>	2.0	2.0	3.3	0	0	0	1.0	1.0	1.8
AE by Severity									
Mild	24.0	17.0	28.3	15	13	21.3	10.0	8.0	14.5
Moderate	17.0	14.0	23.3	15	13	21.3	16.0	12.0	21.8
Severe*	5.0	4.0	6.7	1	1	1.6	5.0	5.0	9.1
SAE by Severity									
Mild	0.0	0.0	0.0	0	0	0	1.0	1.0	1.8
Moderate	2.0	2.0	3.3	2	2	3.3	3.0	3.0	5.5

Severe*	2.0	2.0	3.3	1	1	1.6	1.0	1.0	1.8

Events are counted per occurrence; participants are the number with at least one event; % is the percentage of participants in the group. Related = definitely, possibly, or unknown. Product-relatedness is not applicable to the sham group, which received no product (—). The sham cross-over column counts events after the crossover procedure. *Severe includes events classified as marked. Counts are for all treated participants through the 3-month controlled window, noting that all related events in the randomized arms occurred within 30 days of the procedure.
